# Methodology for the identification of small molecule inhibitors of the Fanconi Anaemia ubiquitin E3 ligase complex

**DOI:** 10.1038/s41598-020-64868-7

**Published:** 2020-05-14

**Authors:** Michael F. Sharp, Vince J. Murphy, Sylvie Van Twest, Winnie Tan, Jennii Lui, Kaylene J. Simpson, Andrew J. Deans, Wayne Crismani

**Affiliations:** 1 St Vincent’s Institute of Medical Research, Fitzroy Victoria, 3065 Australia; 2 Victorian Centre for Functional Genomics, Peter MacCallum Cancer Centre, Melbourne, VIC 3000 Australia; 3 Sir Peter MacCallum Cancer Centre Department of Oncology, University of Melbourne, Melbourne, VIC 3010 Australia; 4 Department of Medicine (St. Vincent’s Health), The University of Melbourne, Melbourne, VIC 3010 Australia

**Keywords:** DNA, Cancer genetics, Biochemistry, Cancer, Cell biology, Drug discovery

## Abstract

DNA inter-strand crosslinks (ICLs) threaten genomic stability by creating a physical barrier to DNA replication and transcription. ICLs can be caused by endogenous reactive metabolites or from chemotherapeutics. ICL repair in humans depends heavily on the Fanconi Anaemia (FA) pathway. A key signalling step of the FA pathway is the mono-ubiquitination of Fanconi Anaemia Complementation Group D2 (FANCD2), which is achieved by the multi-subunit E3 ligase complex. FANCD2 mono-ubiquitination leads to the recruitment of DNA repair proteins to the site of the ICL. The loss of FANCD2 mono-ubiquitination is a common clinical feature of FA patient cells. Therefore, molecules that restore FANCD2 mono-ubiquitination could lead to a potential drug for the management of FA. On the other hand, in some cancers, FANCD2 mono-ubiquitination has been shown to be essential for cell survival. Therefore, inhibition of FANCD2 mono-ubiquitination represents a possible therapeutic strategy for cancer specific killing. We transferred an 11-protein FANCD2 mono-ubiquitination assay to a high-throughput format. We screened 9,067 compounds for both activation and inhibition of the E3 ligase complex. The use of orthogonal assays revealed that candidate compounds acted via non-specific mechanisms. However, our high-throughput biochemical assays demonstrate the feasibility of using sophisticated and robust biochemistry to screen for small molecules that modulate a key step in the FA pathway. The future identification of FA pathway modulators is anticipated to guide future medicinal chemistry projects with drug leads for human disease.

## Introduction

### The significance of FANCD2 mono-ubiquitination in Fanconi anaemia

Bi-allelic germline mutations in one of 22 genes (*FANCA-FANCW*) result in FA, a rare genetic disorder which often leads to progressive bone marrow failure, myelogenous malignancies and squamous cell carcinoma, congenital abnormalities and reduced fertility^1–5^ (with the exceptions of *FANCB*, which is X-linked^6^, and *FANCR/RAD51*, which is autosomal dominant^7^). FA is typically diagnosed with a chromosome breakage test^8,9^. Most FA patients have mutations in *FANC* genes that are required for FANCD2 mono-ubiquitination^10^, to the extent that analysis of FANCD2 mono-ubiquitination in fibroblasts and peripheral blood mononuclear cells is a diagnostic FA assay^11^. Therefore, compounds which can restore FANCD2 mono-ubiquitination could be beneficial to slow the progression of FA-related symptoms. Despite the critical importance of FANCD2 mono-ubiquitination in the biology of FA, recent work has demonstrated that FANCD2 mono-ubiquitination can be uncoupled from nuclear foci formation via the methyl-binding domain of FANCD2 that binds H4K20me2^12^.

There are currently neither systemic and tailored treatments available for FA, nor is there a cure. A recent milestone towards a tailored FA treatment was the successful engraftment of autologous lenti-virus-mediated corrected haematopoietic stem cells in FA patients^13^. This study demonstrates the viability of gene therapy for the haematopoietic system in FA patients, however the elevated cancer risk for the rest of the body^3^ would presumably remain high. Complementary approaches to gene therapy are also being investigated. There are clinical trials with metformin (clinical trials identifier NCT03398824) and quercetin (clinical trials identifier NCT01720147) in progress to identify interventions that could improve manifestations of FA, notably haematological response. TGF-β inhibition is also being investigated as a mechanism of rescue of haematopoiesis in FA^14^. These projects are promising, and they represent a major milestone for research into treatments for FA. However, these small molecule strategies do not specifically target the FA pathway and instead seek to alleviate indirect mechanisms of decreased haematopoiesis in FA; e.g. the presence of ICL-inducing aldehydes or reactive oxygen species. The small molecule trials may eventually be extended to analyse if there is an effect on cancer risk in FA.

## The significance of FANCD2 mono-ubiquitination in malignancies

Increased expression of FANCD2 has been observed in breast and uterine cancers with either *BRCA1/2* alterations or decreased homologous recombination (HR) status^15^. Also FANCD2 expression positively correlates with ovarian carcinoma grade and expression of the proliferative marker Ki-67^15^. Increased FANCD2 expression has also been observed in melanoma^16^. Further, the loss of FANCD2 mono-ubiquitination has been shown to be synthetic lethal with silencing or mutation of *BRCA1*^15^ or *BRCA2*^15,17^. Similarly, simultaneous loss of USP1 – the protein required for deubiquitination of FANCD2^18^ – is synthetic lethal with loss of BRCA1^19^. Further, depletion of FANCI, the binding partner of FANCD2, is also synthetic lethal with silencing or mutation of *BRCA1* or *BRCA2*^15^. Therefore, small molecule-mediated inhibition of the proteins required for the regulated ubiquitination and deubiquitination of FANCD2/FANCI represents a promising strategy to induce cancer-specific killing. There is already clinical precedent for the synthetic lethal approach with PARP inhibitors approved as monotherapies for *BRCA*-mutated cancers – germline or somatic – by the FDA and EMA^20^.

## Molecular biology of FANCD2 mono-ubiquitination

**Figure 1:**
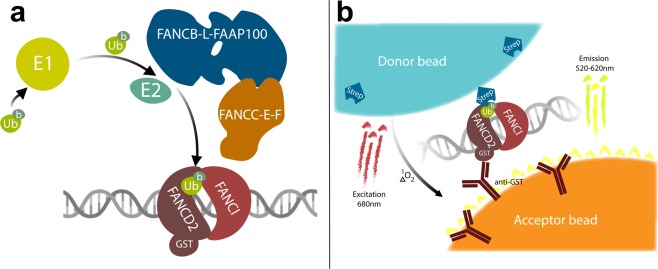
(**a**) Illustration of Fanconi anaemia core-complex mediated ubiquitination of FANCD2 with the 11 proteins used in the inhibitor assay, and (**b**) read out of core complex-mediated ubiquitination of FANCD2 in the proximity assay. When b-ubiquitin is covalently conjugated to GST-FANCD2 they bind the streptavidin-coated donor beads and the anti-GST coated acceptor beads respectively, hence bringing the two bead types into close proximity. When the two bead types are in close proximity – due to FANCD2 mono-ubiquitination – excitation of the donor bead at 680 nm results in the transfer of an oxygen singlet to the acceptor bead. The acceptor bead then emits detectable light between 520–620 nm. Figure created with BioRender.

The FA pathway maintains genomic stability through the repair of ICLs^21,22^ in addition to stabilising stalled replication forks^23,24^ and the removal of DNA-RNA hybrids^25^. One of the first steps of ICL repair via the FA pathway is the coordinated mono-ubiquitination of the heterodimer components FANCD2 and FANCI when bound to DNA^26,27^. FANCD2:FANCI mono-ubiquitination is performed by the “FA core complex”, a multi-subunit E3 ligase complex (Fig. 1a). The core complex is comprised of a ubiquitin E3 RING ligase – FANCL – which is part of a sub-complex with FANCB and FAAP100 (FANC-B-L-100) of stoichiometry 2:2:2^26–30^ and a ubiquitination substrate adaptor composed of FANCC, FANCE and FANCF (FANC-C-E-F). The core complex also contains FANCA, FANCG and FAAP20 (FANC-A-G-20), of which FANCA is mutated in most cases of FA and is required for FANCD2 mono-ubiquitination in cells. FANCA has been shown to be important for single strand annealing, strand exchange^31^ and nuclear import of other core complex components^32^, however no direct biochemical mechanism of FANCA in FANCD2 or FANCI mono-ubiquitination has been demonstrated.

The search for inhibitors of the FA core complex has previously been investigated using genetic, biochemical and cell-free extract approaches^33–38^. A cell-based screen exploited the readout of EGFP-tagged FANCD2 (in a FANCD2-deficient cell line, PD20^39^), which localises to DNA damage after treatment with cisplatin^33^. The absence of FANCD2 foci formation indicated that a compound had interfered with the ability of the FA core complex to ubiquitinate FANCD2, as mono-ubiquitination of FANCD2 was suggested to be required for FANCD2 foci formation^40^. The study identified the natural compound curcumin as the lead hit^33^. The same cell line was used in a similar screen that identified a number of hits that inhibited EGFP-FANCD2 foci formation via mechanisms other than the canonical FA pathway, such as the CHK1 pathway or the proteosome^34^. Another set of investigations found 2,3-dichloro-5,8-dihydroxy-1,4-naphthoquinone and analogs of curcumin as inhibitors of FANCD2 mono-ubiquitination using a *Xenopus* egg extract assay^35,36^. Two different studies have used biochemical approaches to identify inhibitors of the FA pathway. The first biochemical study used a fragment library and a biophysical approach to identify inhibitors of FANCT which resulted in three compounds that were able to inhibit FANCD2 ubiquitination reactions with recombinant proteins. The reaction contained the FANCD2, FANCL and FANCT and the compounds inhibited at 1–4 mM^41^. The second assay used homogenous time-resolved fluorescence to assay for compounds that inhibit auto-ubiquitination of the FANCL RING domain. The auto-ubiquitination was used as a surrogate for FANCD2 mono-ubiquitination and in characterization of the compounds, two hits were found to induce a range of cellular phenotypes consistent with inhibition of FANCD2^37^.

Despite the critical importance of FANCD2 mono-ubiquitination for diagnosing FA and defining the genetic subtypes, there is no reagent which gives a direct read out of only the mono-ubiquitinated or non-ubiquitinated form of FANCD2. Therefore, an antibody raised against FANCD2 is used with low-throughput western blots to measure ratios of mono- and non-ubiquitinated FANCD2, which differ by 8.6 kDa. A reagent which can rapidly and directly measures FANCD2 mono-ubiquitination in patient samples would be a game changer for diagnostics and the ability to screen for drugs that modulate FANCD2 mono-ubiquitination. To overcome the latter and to facilitate drug screening, we have designed a novel high-throughput biochemical compound screen assay (Fig. 1b), which captures much of the complexity of the FA core complex – six of nine FA core complex proteins – plus the heterodimeric FANCD2-FANCI substrate and DNA, which is required for biologically relevant FANCD2 mono-ubiquitination. In total, the assay uses 11 recombinant proteins. This assay has been used to contribute new methodology to probe for activators and inhibitors of the FA core complex.

## Results and Discussion

### Assay development

**Figure 2:**
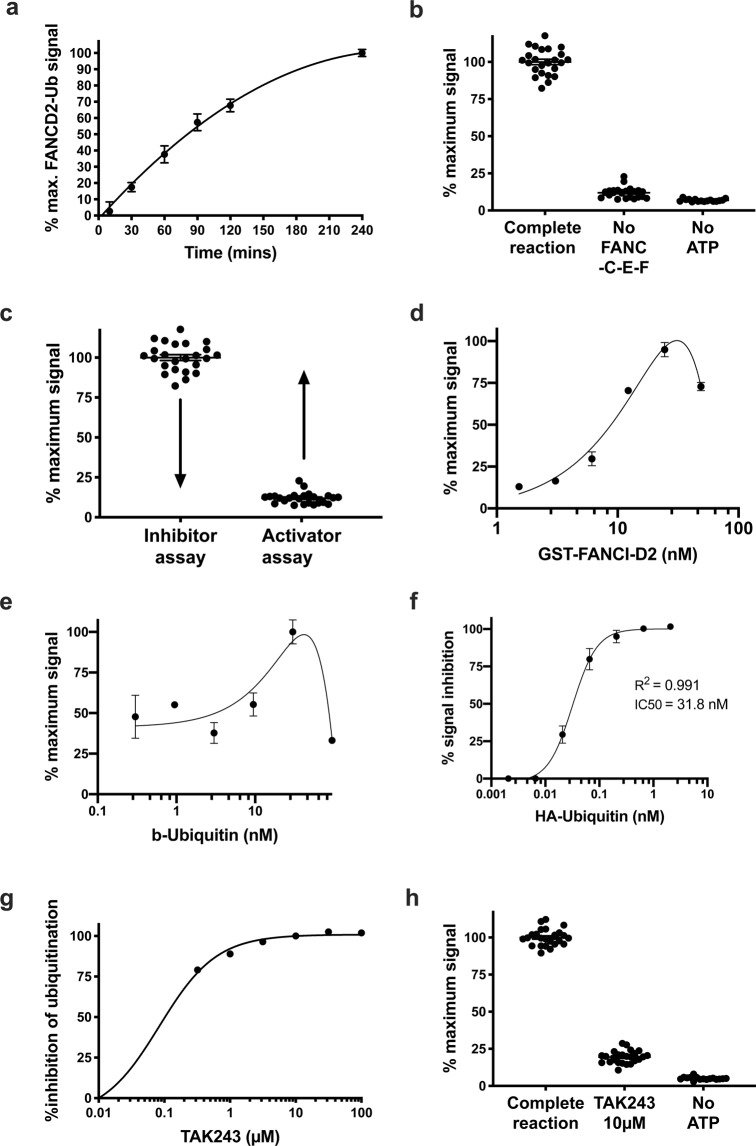
FANCD2-mono-ubiquitination proximity assay parameters. (**a**) Time point ubiquitination assay perform using the proximity assay. (**b**) Demonstration of the controls used for the FANCD2-ubiquitination activator screen. The first condition “complete reaction” represent the FANCD2 mono-ubiquitination reaction with FANCT, FANC-B-L-100, FANC-C-E-F, FANCI, FANCD2 and dsDNA. The second condition and third conditions omit the substrate adaptor FANC-C-E-F (Z′ = 0.57) and ATP respectively. (**c**) Implementation of the two assay conditions to screen for inhibitors and activators of FANCD2 mono-ubiquitination. Arrows highlight that maximal ubiquitination levels will be exploited for the identification of inhibitors and basal levels of ubiquitination will be exploited for the identification of activators. (**d**) Titration of GST-FANCI-D2 complex through the proximity assay. (**e**) Titration of biotinylated-ubiquitin through the FANCD2-ubiquination proximity assay. (**f**) Titration of HA-Ubiquitin through the proximity assay of FANCD2-ubiquination, inhibiting the signal by 50% at a concentration of 31.8 nM. (**g**) Titration of TAK243, which is a ubiquitin-activating-enzyme (UbE1) inhibitor, through the proximity assay. TAK243 is used as an inhibitor control for the inhibitor screen. (**h**) Demonstration of the controls used for the FANCD2-ubiquitination inhibitor screen, Z′ = 0.79.

The inhibitor assay includes recombinant ubiquitin, ubiquitin activating enzyme, FANCT, FANC-B-L-100, FANC-C-E-F, FANCI, FANCD2 and dsDNA^41^ (Fig. 2a). For the activator assay, the activity stimulating adaptor complex FANC-C-E-F is omitted to give a baseline FANCD2-ubiquitination signal which enables a greater dynamic range to detect activation of the E3 ligase sub complex FANCB-L-100 (Fig. 2b,c). These two assays were used to screen 9,067 compounds (Sup. Fig. 1) demonstrating that high-throughput biochemical screens with the FA core complex are possible. The gel-based FANCD2 mono-ubiquitination assay that we published previously^26^ was modified to give a chemi-luminescent readout using a proximity assay (Materials and methods) (Fig. 1b). The purified proteins in the mono-ubiquitination assays used for the high-throughput screen are biotinylated-ubiquitin (b-ubiquitin), His-UBE1, FANCT, Flag-FANCB, FANCL, FAAP100, MBP-FANCC, FANCE, FANCF, GST-*X*lFANCD2 and Flag-*Xl*FANCI. The assay was calibrated for maximal signal to background and for an optimal dynamic range within which small molecule modulators could be detected in a 384-well plate format. A number of variables were tested, notably including (1) the amounts of GST-FANCD2 (Fig. 2d) and b-ubiquitin (Fig. 2e), (2) the amount of core complex to GST-FANCD2, and (3) the incubation time with the beads (Fig. 2a). The addition of the beads also stops the mono-ubiquitination reaction, most likely due to steric hindrance given the greater size of the beads compared to the GST-FANCD2 and b-ubiquitin.

In order to attribute the 520–620 nm emission from the assay to ubiquitination of FANCD2, a competition assay using HA-tagged ubiquitin that cannot bind the donor beads, was performed. This was achieved with the titration of HA-ubiquitin through the proximity assay (Fig. 2f). 31.8 nM of HA-Ubiquitin was required to inhibit 50% of the signal generated from 30 nM of biotinylated ubiquitin (R^2^ = 0.991), demonstrating that the signal emitted at 520–620 nm is due to the conjugation of ubiquitin onto FANCD2.

### FANCD2 mono-ubiquitination activator screen

**Figure 3:**
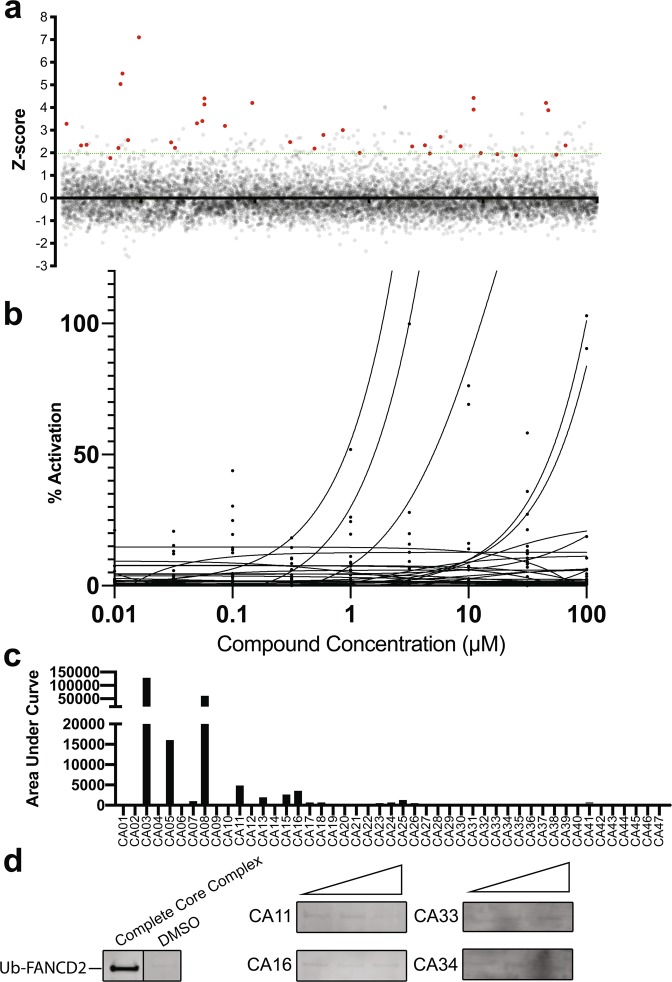
Primary screen for activators of FANCD2 mono-ubiquitination in the absence of FANC-C -E -F. (**a**) All compounds screened in duplicate plotted as Z scores and presented on a per library representation. (**b**) Nine-point titration of 47 activator hits. (**c**) Area under curve plot calculated from the curves in 3b, demonstrating activation potency for each compound. (**d**) Cropped images from western blot analysis of 4 activator hits for an effect on the FANCD2 mono-ubiquitination assay with recombinant protein. Positive (Complete Core Complex) and negative (DMSO) controls are shown on the far-left panel. The four activator hits from the secondary screen were analysed with FANCD2-ubiquitination assay (without FANC-E-F) visualised on western blot by probing with streptavidin to detect biotinylated-ubiquitin. Three concentrations of the compounds were used; 1 µM, 10 µM and 100 µM.

Currently there is no specific treatment for FA that directly targets the biochemical cause of the condition. More than 90% of FA patients mutations occur in genes with established roles in FANCD2 mono-ubiquitination^9,10,42^. These mutations typically result in a reduction or loss of FANCD2 mono-ubiquitination and defective repair of ICLs^6,11,43–47^. We performed a high throughput screen for small molecule activators of the FANCD2 mono-ubiquitination, which lacks the substrate adaptor (FANC-C-E-F) (Fig. 2b,c). The 9,067 compounds tested (see Materials and Methods) in the activator assay were screened in duplicate and hits were selected at a Z-score cut off of >2 (FDR = 0.0025). Forty-five compounds produced a Z-score >2 and were investigated further (Fig. 3a). We used the substrate adaptor (FANC-C-E-F) protein complex as the most appropriate available positive control as there is no small molecule activator of FANCD2 mono-ubiquitination available. These preliminary hits were counter-screened by testing for interference with the excitation-emission properties of the beads, as opposed to modulation of FANCD2 mono-ubiquitination used in the assay (Sup. Fig. 2a). This bead interference assay was performed by incubating these compounds with biotinylated-GST and the proximity assay beads. Following the primary screen, putative hit compounds were next analysed over a 9-point dose response curve and quantified using the proximity assay (Fig. 3b,c). The FDA-approved compound subset contained some blue coloured compounds which generated signals many-fold higher than the FANCC-E-F positive controls. These were excluded as false positive activators (Sup. Fig. 2a). Only four compounds (CA11, CA16, CA33 and CA34) showed weak concentration-dependent activation using the proximity assay without false-positive activation in the bead interference assay. These four compounds were tested by western blot and none were shown to increase FANCD2 ubiquitination compared to controls (Fig. 3d). While we did not identify any compounds that increased the levels of FANCD2 mono-ubiquitination, we have demonstrated that it is possible to perform high-throughput activator screens with biochemical approaches using the direct target that is defective in nearly all FA patients. Subsequent iterations of this activator screen may benefit from screening all the scaffold compounds which would enable extensive structure-activity-relationship analysis to be performed that may identify an activating chemotype. Similarly, future approaches to find activators of FANCD2 mono-ubiquitination with this assay may include patient mutant variants of FANC-C-E-F or the addition of FANC-A-G-20. However, FANC-A-G-20 was not included in this screen as it has no detectable effect on FANCD2 mono-ubiquitination in our reconstituted biochemical system^26^.

### FANCD2 mono-ubiquitination inhibitor screen

Prior to performing the inhibitor screen, we identified a positive control small molecule inhibitor of the recombinant FANCD2 mono-ubiquitination reaction. The compound TAK243 is a small molecule that covalently binds to the C-terminus of ubiquitin and occupies the adenylate (AMP)-binding site of UBE1^38^. TAK243 was a highly potent inhibitor of the FANCD2 mono-ubiquitination reaction with >50% inhibition at 0.3 µM (Fig. 2g,h).

**Figure 4:**
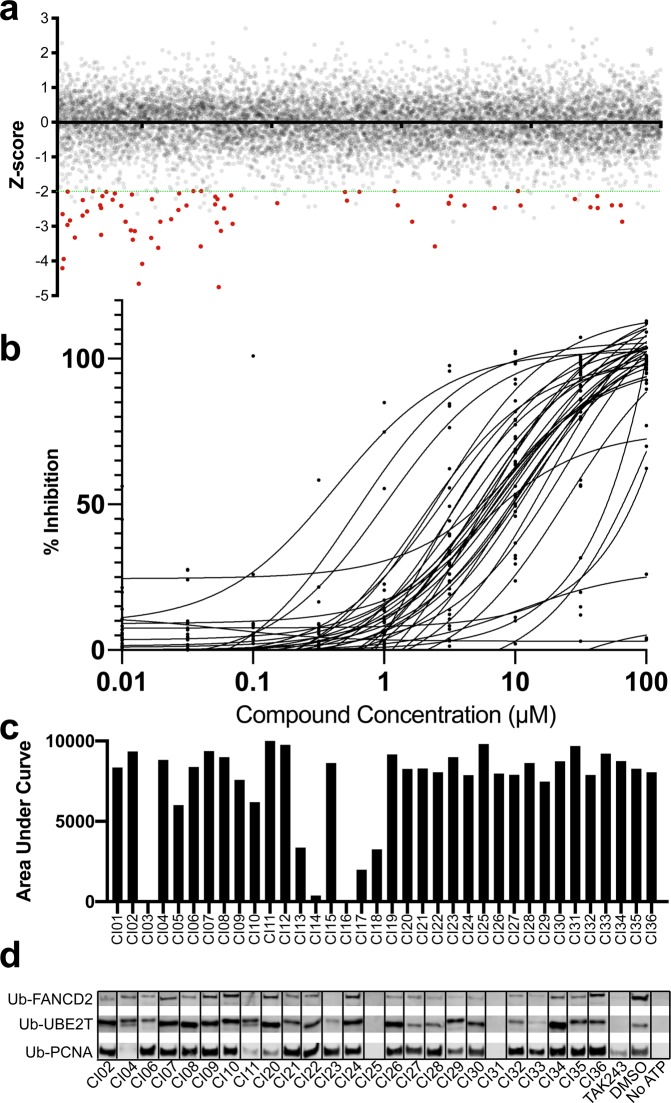
Primary screen for inhibitors of FANCD2 mono-ubiquitination. (**a**) All compounds screened in duplicate plotted as z-scores and presented on a per library representation. (**b**) Nine-point titration of 36 inhibitor hits. (**c**) Area under curve plot calculated from the curves in 4b, demonstrating activation potency for each compound. (**d**) Cropped images from western blot analysis of 32 inhibitor hits for an effect on the FANCD2 mono-ubiquitination assay with recombinant protein. The hits were analysed with FANCD2-ubiquitination assay visualised on western blot by probing with streptavidin to detect biotinylated-ubiquitin.

We performed a high throughput screen for inhibitors of FANCD2 mono-ubiquitination with the proximity assay using the same 9,067 small molecules screened in duplicate in the activator screen. Thirty-six compounds with a Z-score < −2 and were selected for further investigation (Fig. 4a). Similar to the activator screen, these preliminary hits were counter-screened testing for interference with the bead assay (Sup. Fig. 2b) and over a 9-point dose response (Fig. 4b,c, Sup. Table 1). Compounds that showed dose-dependent inhibition of FANCD2 mono-ubiquitination greater than their effect on bead interference were then analysed for inhibition of FANCD2 mono-ubiquitination by western blot (Fig. 4d, Sup. Fig. 4). Twenty-five compounds were tested (Sup. Fig. 1), and 19 inhibited (>80% compared to DMSO) FANCD2 mono-ubiquitination. Therefore, we sought to test for specificity of these compounds for the FA pathway by investigating if the highest ranking 25 compounds inhibited another recombinant ubiquitination reaction. We tested the same 25 compounds for inhibition of mono-ubiquitination of PCNA by UbcH5c (Fig. 4d, Sup. Fig. 4). Eighteen compounds did not inhibit PCNA ubiquitination (≤ 0% compared to DMSO). In total, 13 compounds inhibited FANCD2 mono-ubiquitination (> 80%) but had no effect on PCNA ubiquitination.

### Cell-based assays

**Figure 5:**
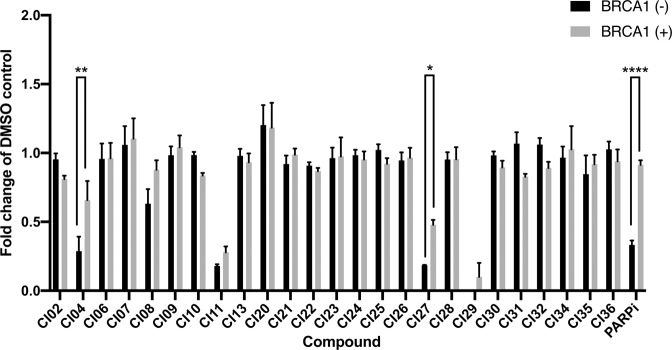
Synthetic lethality ability of the 25 lead compounds. Synthetic lethality was assessed by performing a SRB Cell viability assay on SUM149 parental (*BRCA1*-negative) and SUM149 (BRCA1 reversion) in the presence of 25 lead inhibiting compounds at a concentration of 10 µM. * = P < 0.05, ** = P < 0.01, ** = < 0.0001, n = 3.

The twenty-five compounds that appeared to selectively inhibit the FANCD2 mono-ubiquitination proximity assay were next tested in cell-based assays. The simultaneous loss of FANCD2 or FANCD2 ubiquitination with *BRCA1* deficiency has been shown to be synthetic lethal^15^. Therefore, we tested our preliminary inhibitors for the ability to kill *BRCA1*-deficient cells preferentially over isogenic *BRCA1-*proficient controls. To achieve this we exploited the triple negative breast cancer cell line SUM149^48^ with a homozygous loss of function mutation in *BRCA1* (c.2288delT, p.N723fsX13) and the daughter cell line, SUM149.B1.S* with one allele containing a secondary restoration of function mutation (an 80-bp BRCA1 deletion, c.[2288delT;2293del80])^49^. Twenty-five compounds were tested at 10 µM for preferential cytotoxicity in the *BRCA1*-deficient SUM149 (Fig. 5). The positive control compound, the PARP inhibitor olaparib, preferentially killed SUM149 cells over SUM149.B1.S* (33.3% vs 78.1%). Two compounds, showed a preferential killing effect of *BRCA1*-negative SUM149 cells, despite also showing toxicity in the B1.S* control at the concentration (10 µM) tested (CI04, *p* = 0.001 [SUM149, 28.7% survival vs SUM149 B1.S*, 55.7% survival], CI27 *p* = 0.011 [SUM149, 18.4% survival vs SUM149 B1.S*, 43.7% survival]). However, CI27 did not reduce FANCD2 mono-ubiquitination in cells as measured by western blot (Sup. Fig. 3), suggesting that any preferential killing of *BRCA1*-deficient cells is not likely to be via specific inhibition of the FA core complex. CI04 was not investigated by western blot of cell extract as it did not specifically inhibit FANCD2 mono-ubiquitination based on the PCNA ubiquitination assay. The combination of the FANCD2, PCNA and cell-based assays revealed 11 compounds that might specifically inhibit FANCD2 mono-ubiquitination, or that at least are not pan ubiquitination inhibitors.

### FANCD2 foci formation

**Figure 6:**
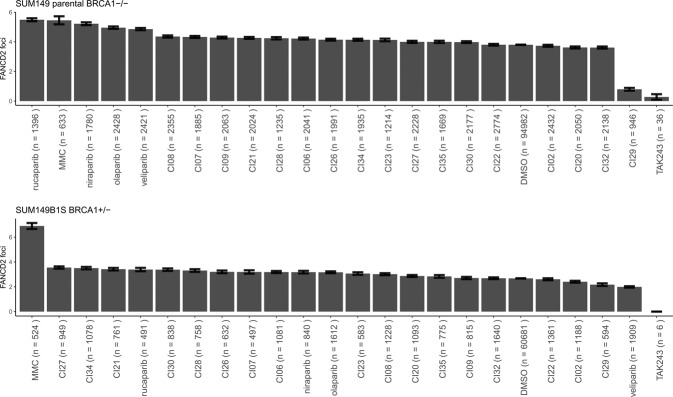
FANCD2 foci. FA pathway activity was assayed by immunofluorescence of FANCD2 foci. 11 compounds (1 µM) identified from the inhibitor screen were analysed.

FANCD2 foci formation is often used as an indicator of FA core complex functionality^40,43^. Therefore, we selected 11 compounds to test at 1 µM in 384-well format for their ability to reduce FANCD2 foci formation in SUM149 parental *BRCA1-*deficient cells. These 11 compounds were selected (Sup. Fig. 1) because they demonstrated a potential specific reduction in FANCD2 mono-ubiquitination or preferentially killed *BRCA1*-deficient cells. None of the 11 compounds (1 µM) significantly reduced FANCD2 foci formation, however positive control TAK243 did inhibit FANCD2 foci formation in SUM149 parental cells (TAK243 = 0.28 v DMSO = 3.80, *p* < 0.0001), while being highly toxic. We also used mitomycin C, which is known to result in increased FANCD2 foci formation^40^, and this was true for SUM149 parental cells (MMC = 5.45 v DMSO = 3.80, *p* < 0.0001). The increase in FANCD2 foci in the presence of mitomycin C validates this cell-based assay (Fig. 6) and helps to establish TAK243 as a control inhibitor for FANCD2 mono-ubiquitination both with recombinant protein and in cells^50^. We included four effective PARP inhibitors, veliparib, rucaparib, olaparib and niraparib, to assess their effect on FANCD2 foci formation and found all increased FANCD2 foci formation in SUM149 parental cells (*BRCA1-*deficient) but not in SUM149 B1.S* (with one functional copy of *BRCA1*) (Fig. 6). The PARP inhibitor-dependent increase in FANCD2 foci formation suggests that these compounds increase the reliance of cells on the canonical FA pathway in these *BRCA1-*deficient cells, but not their isogenic *BRCA1*-proficient counterparts. An inhibitor of the FA pathway could exploit this dependency of FANCD2 activity to synergise with PARP inhibitors.

In summary this study shows a new tool for high-throughput screening with a functional reconstituted FA core complex. In the future, the development of targeted therapies may well benefit from the use of this screening tool when coupled with testing of diverse compound libraries and targeted molecules guided by structural data for the human FA core complex and FANCD2-FANCI.

## Methods

### Recombinant FA core complex

#### Cloning of MultiBAC expression vectors

*Xenopus laevis* FANCD2 was cloned into pDEST20-GST, resulting in a N-terminal tagged GST-FANCD2 and was co-purified with *Xl*FANCI. The other proteins were human proteins and the cloning of the relevant plasmids was described previously^26,51,52^.

### Protein production and purification

FANC-B-L-100 complex purification: High-Five cells were infected with BL100 virus at MOI of 2 and after 72 hrs were harvested by centrifugation at 500 g for 5 mins. Cells were resuspended in buffer A (20 mM Triethanolamine pH 7.5, 10% glycerol, 150 mM NaCl, 1 mM EDTA, mammalian protease inhibitors (Sigma P8340)). Cells were lysed by sonication and lysates were centrifuged at 18,000 g for 30 mins. M2 ANTI-FLAG resin (Sigma A2220–10ML) was washed with 5 column volumes of buffer B (20 mM Triethanolamine pH 7.5, 10% glycerol, 150 mM NaCl) then 3 column volumes of 100 mM Glycine pH 3.5 and equilibrated with 5 column volumes of buffer B. The clarified lysate was added to the resin and incubated at 4 °C for 90 mins with gentle mixing. Resin was then washed with buffer B and FANC-B-L-100 was eluted in 1 column volume of 100 µg/ml FLAG peptide in buffer B at a concentration of 0.5 mg/ml.

FANCI and FANCD2 complex purification: High-Five cells were co-infected with FLAG-FANCI and GST-FANCD2 viruses each with a MOI of 1 and after 72 hrs were harvested by centrifugation at 500 g for 5 mins. Cells were resuspended in buffer and after sonication lysates were centrifuged at 18,000 g for 30 mins. Glutathione sepharose 4B resin (GE Healthcare, 17–0756–01) was equilibrated with buffer B according to the manufacturer’s instructions. Clarified lysate was added to the glutathione sepharose resin and incubated at 4 °C for 90 mins with gentle mixing. Sepharose was washed with 25 column volumes of buffer B. Elution was performed with 3 column volumes of buffer B with 40 mM reduced glutathione. Protein was then loaded onto equilibrated M2 flag resin and cycled through the column for 30 mins at 4 °C. Resin was washed with buffer B and FANCD2-FANCI heterodimer was eluted with 1 ml fractions of 100 µg/mL FLAG peptide in buffer B. Fractions containing ID2 were pooled and aliquoted. Protein concentration was ~0.3 mg/ml. FAN-C-E-F complex purification was described previously^26^. UBE2T/FANCT purification: Steps were carried out at 4 °C. BL21 (DE3) *E. coli* cells expressing UBE2T (15 g paste) were lysed in 75 mL of CBB buffer containing 500 mM KCl with 0.1% IGEPAL, sonicated (3 ×5 mins Branson 450 power setting 2) and clarified (40,000 g x 30 mins). The cleared lysate was diluted with an equal volume of T buffer (25 mM Tris-HCl, 10% glycerol, 0.5 mM EDTA, 0.01% IGEPAL, 1 mM DΤΤ, and protease inhibitors) with 300 mM KCl and then mixed with 3 mL Glutathione-Sepharose beads for 1.5 hours. The affinity beads were washed with 50 mL each of T buffer with 500 mM KCl and T buffer with 1 M KCl, before being treated with 18 mL T buffer with 100 mM KCl and PreScission protease overnight 4 °C. The eluate was passed through a 120 uL complete His Tag resin (Roche) equilibrated with T buffer with 100 mM KCl to remove PreScission protease. The cleaved GST-UBE2T was passed over fresh Glutathione-Sepharose beads to ensure removal of GST-Ube2T or free GST. Ubiquitin with an N-terminal-conjugated biotin was produced with one of two methods, both which produced similar results. The first method is described previously^52^ or with the following method: Ubiquitin (Boston Biochemicals) was dissolved in water to give 10 mg/ml solution. EZ-L (EZ-Link-Silfo-NHS-Biotin (EZ-L) Thermo) was dissolved in dry DMSO (Molecular Probes) to give 10 mg/ml. One mL of ubiquitin solution was diluted with 30 mL 0.1 M NaPO4, pH 3.5 and added to 2 mL EZ-L solution incubated overnight at 37 °C. The reaction was stopped with 2 mL 1 M Trifluoroacetic acid (TFA). Reaction products were separated using HPLC with a Vydac C18 10 ×250 (218TP510 Vydac) column with a 40–80% buffer B gradient for 40 minutes at 2.5 mL/min. Buffer A was 0.1% TFA in water. Buffer B was 0.1% TFA in 70% acetonitrile. Peaks were collected and submitted for MS/MS analysis to isolate the fraction with N-terminal biotinylated ubiquitin.

### Mono-ubiquitination proximity assay and compound libraries

For high-throughput screening, FANCD2 mono-ubiquitination was measured with a proximity assay (AlphaScreen, Perkin Elmer). The donor and acceptor beads are coated in streptavidin and anti-GST respectively. Therefore, when biotinylated ubiquitin is conjugated onto GST-FANCD2 this brings the two beads into close proximity. Light at 680 nm is used to excite the donor bead, which transfers an oxygen singlet to the acceptor bead which emits detectable light at 520–620 nm. The 520–620 nm signal provides a read out of FANCD2 mono-ubiquitination. The ubiquitination proximity assays were performed in 7 µL reactions in 384-well AlphaPlates (Perkin Elmer). A master mix was prepared for ubiquitination assay. The inhibitor screen master mix contained 30 nM biotinylated ubiquitin, 9.1 nM ubiquitin activating enzyme (Boston Biochem), 94.5 nM of FANCT, 86 nM FANC-B-L-100 complex, 25 nM FANCD2-FANCI heterodimer, 93.1 nM FANC-C-E-F complex, 2.4 ng/mL 60 bp dsDNA. Five microlitres of master mix was incubated with 15 nL of a 5 mM compound stock (Compounds Australia, Griffith University) in DMSO, which corresponded to a final concentration of 0.2% DMSO. A total of 9,067 compounds were selected from two libraries, described previously^53,54^, from Compounds Australia. The first library – the Open Access Drug library – contained 2,667 compounds of primarily FDA-approved drugs. The second library, the Open Collection Scaffold library, was used to screen a selection of 6,400 compounds representing approximately 1,000 scaffold bases. All protein concentrations in the assay were determined by titration of each component for optimal activity (Fig. 2). Reaction mixtures were left to incubate with compound for 30 minutes before the reaction was started by adding ATP to a final concentration of 1.14 mM. The FANC-C-E-F complex was omitted from the activator screen mastermix. The inhibitor and activator screen reactions proceeded for 90 mins and 240 mins, respectively. Reactions were stopped by adding a 5 µL working solutions of streptavidin donor beads, immediately followed by 5 µL of anti-GST acceptor beads (Perkin Elmer), each at a final concentration of 0.06%. The controls for the inhibitor assay were 0.2% DMSO (vehicle), 0.2% DMSO plus 10 µM TAK243 ubiquitin activating enzyme inhibitor (positive control inhibitor) and no ATP (control for background signal). The activator assay controls were 0.2% DMSO (vehicle), 0.2% DMSO plus FANC-C-E-F (positive control activator) and no ATP (control for background signal). All of the liquid handling steps were performed robotically on a Sciclone ALH 300 (Caliper Life Sciences, Perkin Elmer). The ubiquitination master mixes, ATP working stock and acceptor and donor bead solutions were prepared in 384 deep-well master plates, in which respective volumes were transferred from these master plates to an assay plate using the Sciclone ALH300 to reduce reagent wastage. Between four and 11 assay plates were performed at a time with 160 compounds on each plate. All compounds in the primary screen were analysed in duplicate. Plate columns 1, 2 and 23 contained DMSO and activator and inhibitor controls. Column 24 contain No ATP. Data from each plate was normalised to using moving average z-scores (z-score = (sample raw score – population mean)/population standard deviation). Moving average was calculated using a radius of four wells around each sample to account for plate and edge effects. Activating and inhibiting compounds to carry forward to secondary screening were defined as those that had less than 30% variance between duplicates had z-scores greater than 2 or less than −2, respectively. A 9-point dose response was performed for secondary screening using a half-logarithmic dilution series. Percentage inhibition and activation was calculated for respective dose-responses and compounds were ranked by area-under the curve calculations (AUC). AUC was used instead of IC50/AC50 as some compounds demonstrated inhibition or activation but did not reach 50% inhibition or activation within this concentration range tested. IC50 values and R^2^ values are in Sup. Table 1. Plates were read on an Enspire (Perkin Elmer).

### Cell lines and culture conditions

SUM149 parental cells and SUM149 B1.S* cells^49^ were cultured in Hams-F12 from Gibco supplemented with 10% fetal bovine serum, 0.24 IU/mL of Insulin, 50 ng/mL Hydrocortisone and 1X penicillin/streptomycin. Both cells lines were grown at 37 °C in a 5% CO_2_ atmosphere. Insect cell lines. High Five cells (Invitrogen) were cultured in Sf-900II SFM media from Gibco and were incubated at 27 °C, shaking.

### Synthetic lethal cell assay

SUM149 parental cell line and SUM149 B1.S* cell lines^49^ were seeded in a 96-well plate with 3000 cells per well. After 24 h, media was removed, and the cells were treated with 10 µM of each compound in triplicate in antibiotic free media and left for 72 h. Cell were fixed with 1% trichloroacetic acid and stained with sulphorhodamine B (SRB). Absorbance of wells were read at 550 nm.

### Detection of nuclear FANCD2 foci

Nuclear FANCD2 foci were measured in high throughput by quantitating DAPI stained cell nuclei of either SUM149 or SUM149 B1.S* cell lines using automated confocal imaging (CX7 LZR, ThermoFisher Scientific). Control DMSO, treated cells reached 85% confluence at the conclusion of 2 days of 1 µM drug exposure. FANCD2 foci were detected with immunofluorescence using rabbit anti-human FANCD2 (abcam, ab108928). 1 µM was selected in order to have sufficient nuclei for FANCD2 foci quantification, as 10 µM compounds was too toxic for certain compounds.

## Supplementray information

Supplementray information1.
Supplementray information2.
