# Supplementary material for: Methodology for the identification of small molecule inhibitors of the Fanconi Anaemia ubiquitin E3 ligase complex
Source: Sci Rep. 2020 May 14;10:7959. doi: 10.1038/s41598-020-64868-7 (PMC7224301; doi:10.1038/s41598-020-64868-7)
Supplement: Supplementary file 1 — Supplementray information1. [file 41598_2020_64868_MOESM1_ESM.pdf]

Supplementary Information

Methodology for the identification of small molecule inhibitors of the Fanconi Anaemia ubiquitin E3 ligase complex

Authors: Michael F. Sharp[1], Vince J. Murphy[1], Sylvie Van Twest[1], Winnie Tan[1,4], Jennii Lui[2], Kaylene J. Simpson[2,3], Andrew J. Deans[1,4], Wayne Crismani[1,4]

Author affiliations:

1. St Vincent's Institute of Medical Research, Fitzroy, Victoria, Australia
2. Victorian Centre for Functional Genomics, Peter MacCallum Cancer Centre, Melbourne, VIC 3000, Australia
3. Sir Peter MacCallum Cancer Centre Department of Oncology, University of Melbourne, Melbourne, VIC 3010, Australia
4. Department of Medicine (St. Vincent's Health), The University of Melbourne, VIC 3010.

Corresponding author: wcrismani@svi.edu.au

Biotinylated-Ubiquitin IR800 Fig 3d

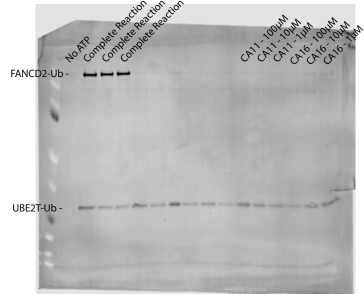

Biotinylated-Ubiquitin IR800 Fig 3d

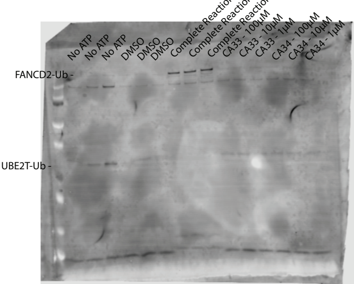

biotinylated-ubiquitin IR800 Fig 4d

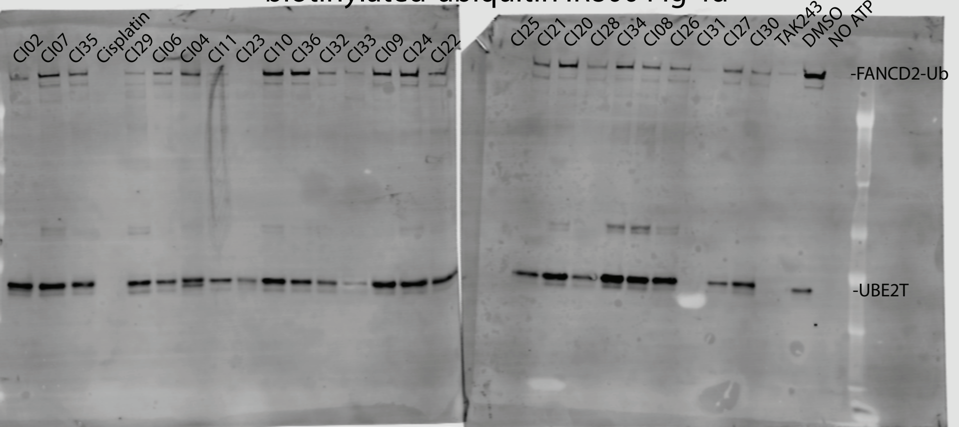

Anti-PCNA IR800 - (Fig 4d)

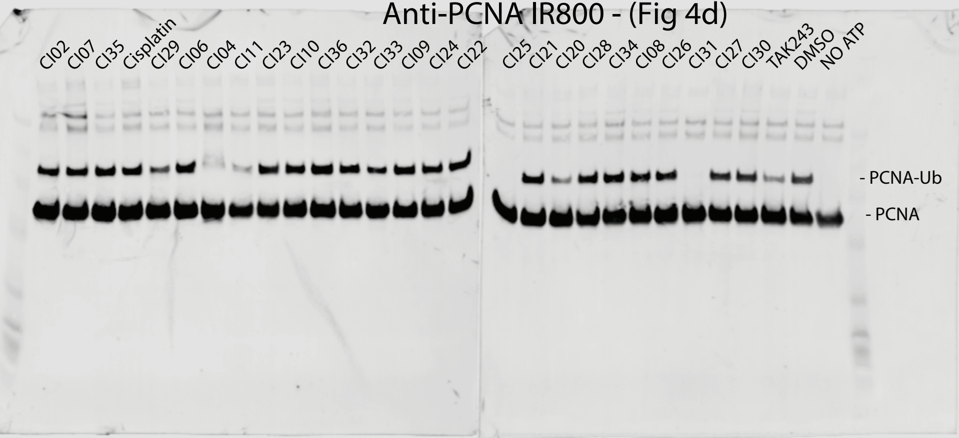

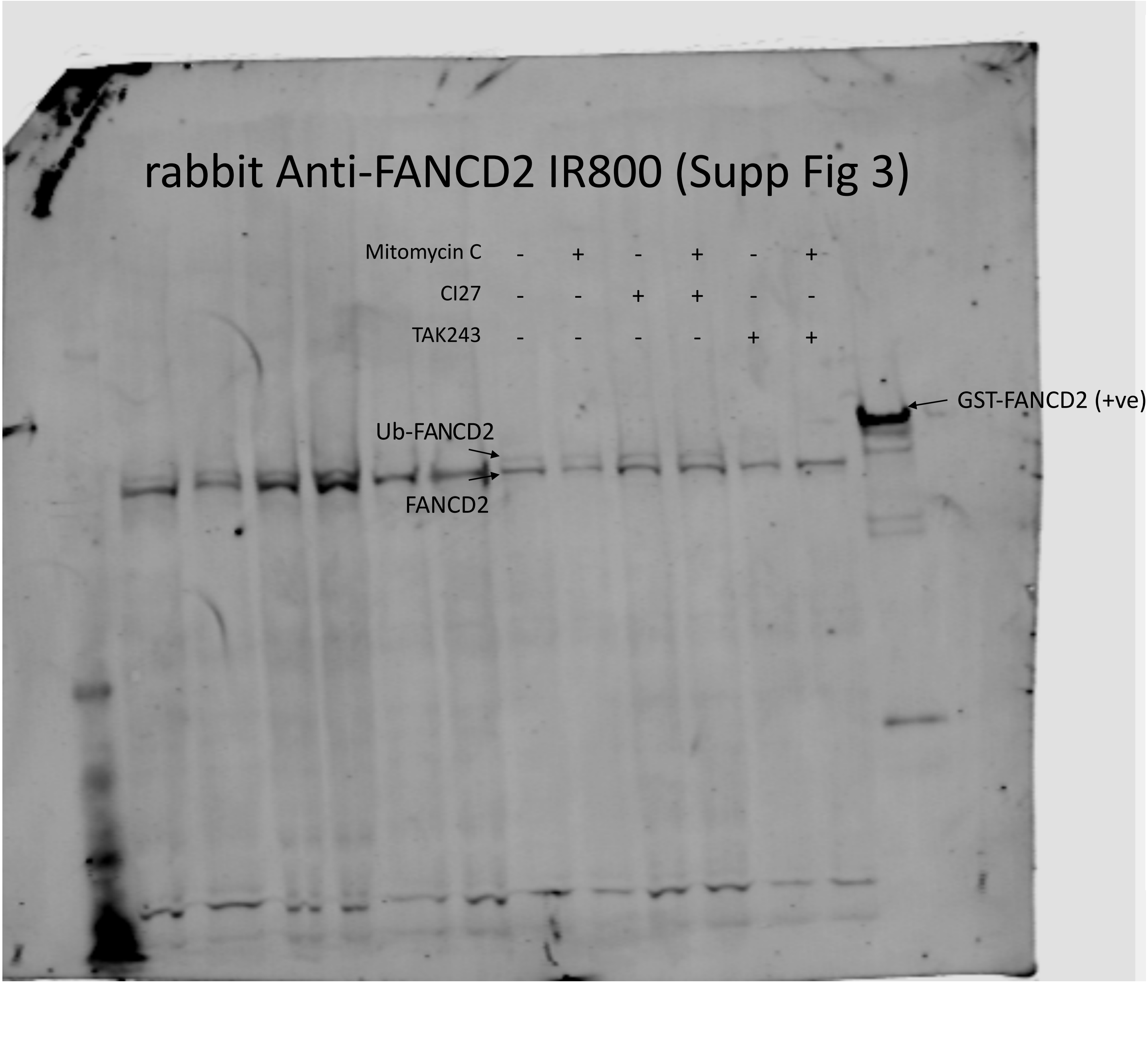

rabbit Anti-FANCD2 IR800 (Supp Fig 3)
Mitomycin C - + - + - +
CI27 - - + + - -
TAK243 - - - - + +
GST-FANCD2 (+ve)
Ub-FANCD2
FANCD2
