# Supplementary material for: Methodology for the identification of small molecule inhibitors of the Fanconi Anaemia ubiquitin E3 ligase complex
Source: Sci Rep. 2020 May 14;10:7959. doi: 10.1038/s41598-020-64868-7 (PMC7224301; doi:10.1038/s41598-020-64868-7)
Supplement: Supplementary file 2 — Supplementray information2. [file 41598_2020_64868_MOESM2_ESM.pdf]

Supplementary Information

Methodology for the identification of small molecule inhibitors of the Fanconi Anaemia ubiquitin E3 ligase complex

Authors: Michael F. Sharp[1], Vince J. Murphy[1], Sylvie Van Twest[1], Winnie Tan[1,4], Jennii Lui[2], Kaylene J. Simpson[2,3], Andrew J. Deans[1,4], Wayne Crismani[1,4]

Author affiliations:

1. St Vincent's Institute of Medical Research, Fitzroy, Victoria, Australia
2. Victorian Centre for Functional Genomics, Peter MacCallum Cancer Centre, Melbourne, VIC 3000, Australia
3. Sir Peter MacCallum Cancer Centre Department of Oncology, University of Melbourne, Melbourne, VIC 3010, Australia
4. Department of Medicine (St. Vincent's Health), The University of Melbourne, VIC 3010.

Corresponding author: wcrismani@svi.edu.au

Sup. Fig. 1

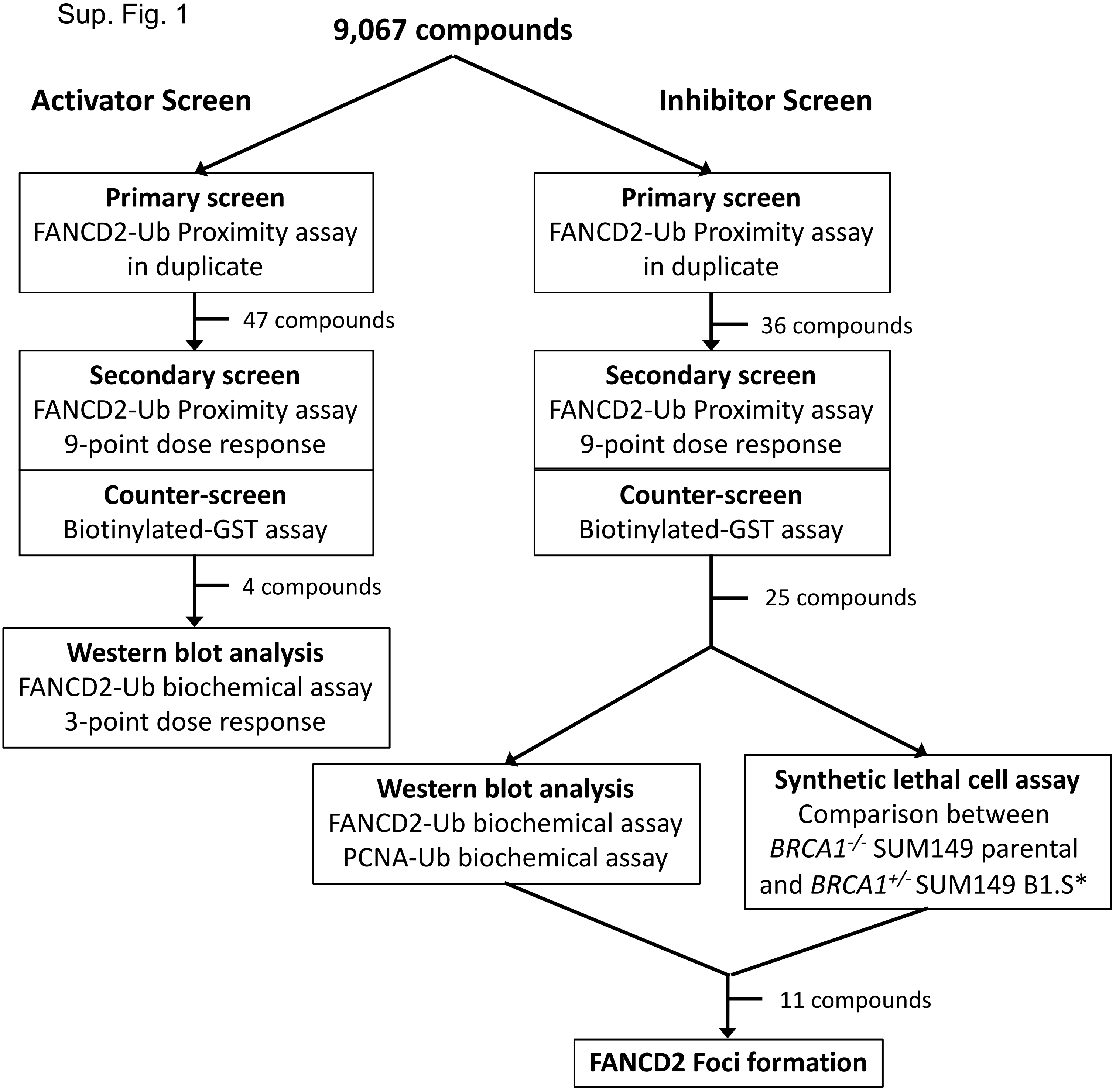
9,067 compounds
Activator Screen
Inhibitor Screen
Primary screen
FANCD2-Ub Proximity assay
in duplicate
47 compounds
Secondary screen
FANCD2-Ub Proximity assay
9-point dose response
Counter-screen
Biotinylated-GST assay
4 compounds
Western blot analysis
FANCD2-Ub biochemical assay
3-point dose response
Primary screen
FANCD2-Ub Proximity assay
in duplicate
36 compounds
Secondary screen
FANCD2-Ub Proximity assay
9-point dose response
Counter-screen
Biotinylated-GST assay
25 compounds
Western blot analysis
FANCD2-Ub biochemical assay
PCNA-Ub biochemical assay
Synthetic lethal cell assay
Comparison between
BRCA1$^{-/-}$ SUM149 parental
and *BRCA1*$^{+/-}$ SUM149 B1.S*
11 compounds
FANCD2 Foci formation

Sup. Fig. 2

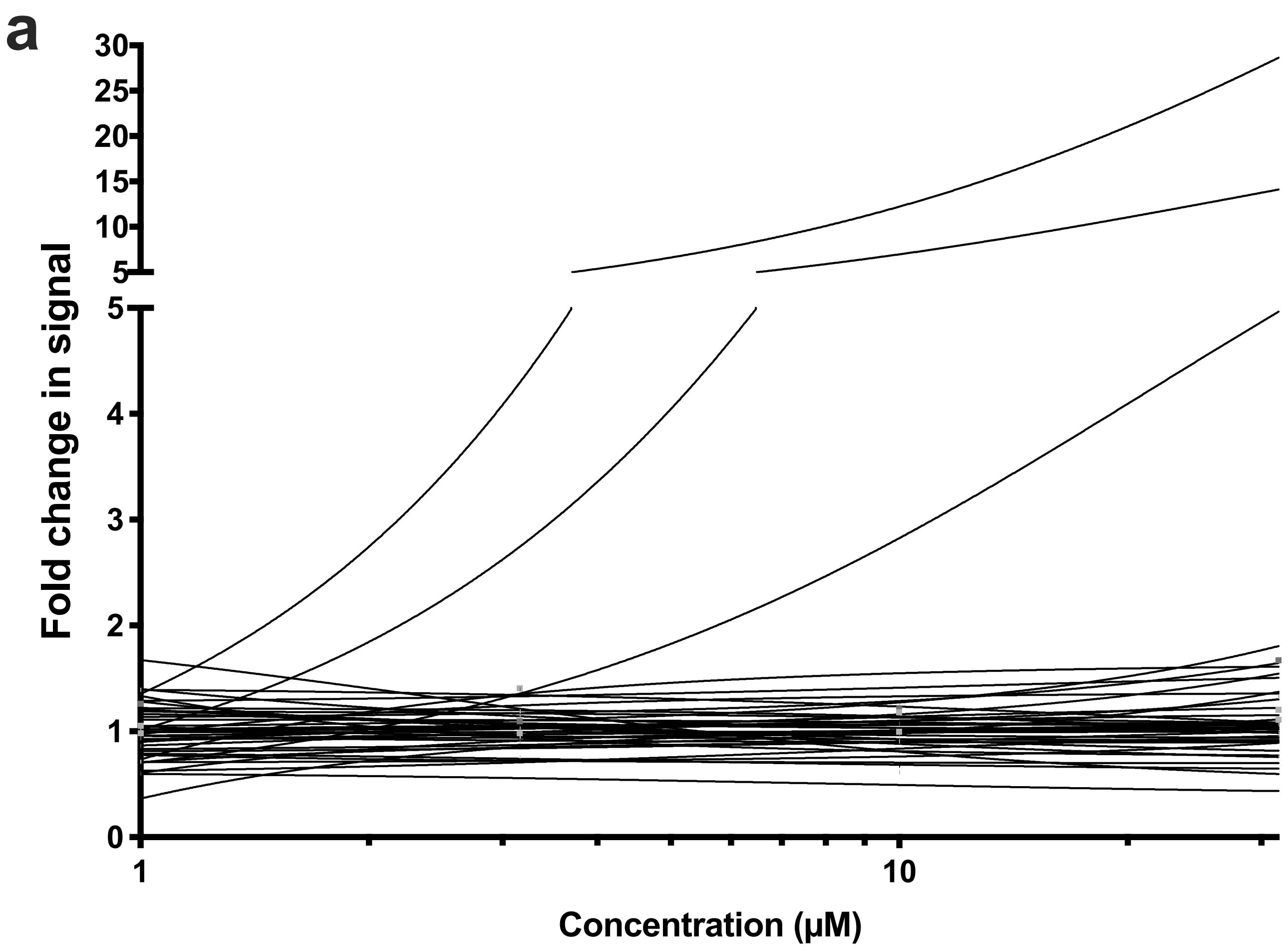
a
30
25
20
15
10
5
5
4
3
2
1
0
Fold change in signal
1
10
Concentration (µM)

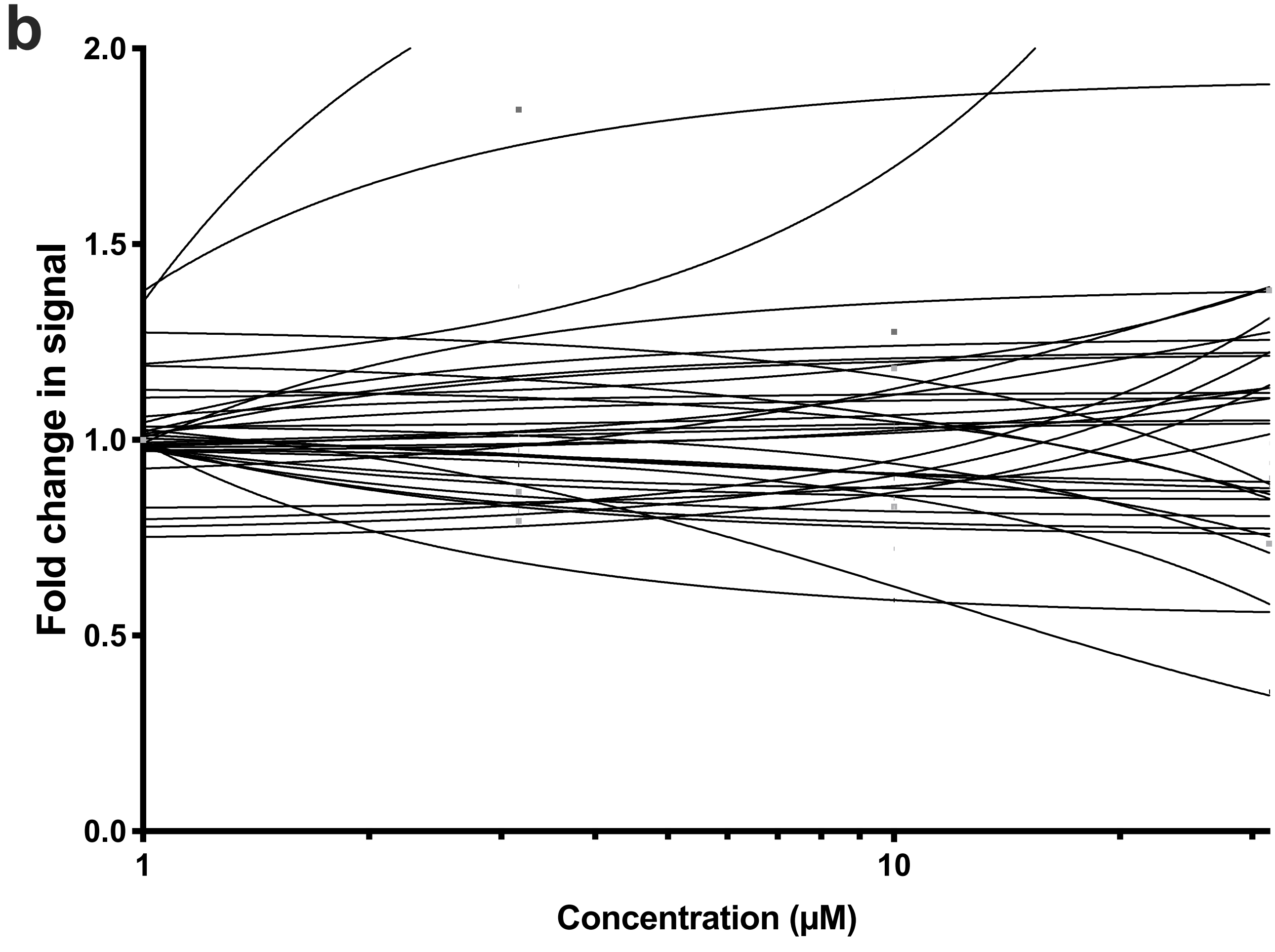
b
2.0
1.5
1.0
0.5
0.0
Fold change in signal
1
10
Concentration (µM)

Sup. Fig. 3

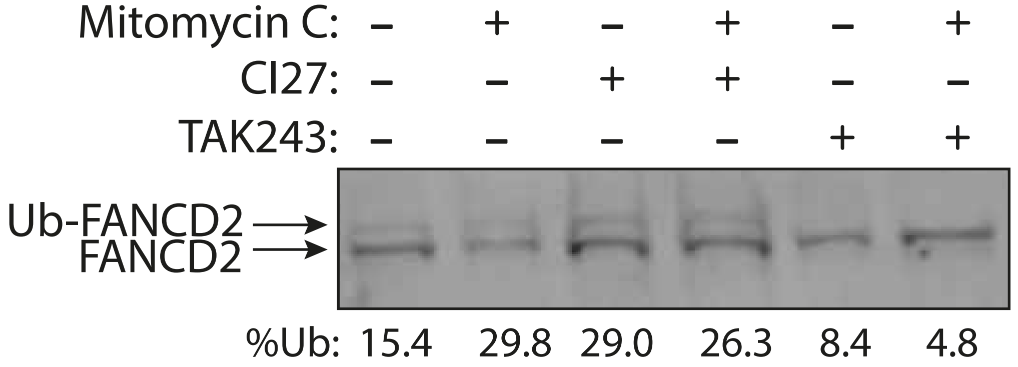
Mitomycin C: – + – + – +
CI27: – – + + – –
TAK243: – – – – + +
Ub-FANCD2
FANCD2
%Ub: 15.4 29.8 29.0 26.3 8.4 4.8

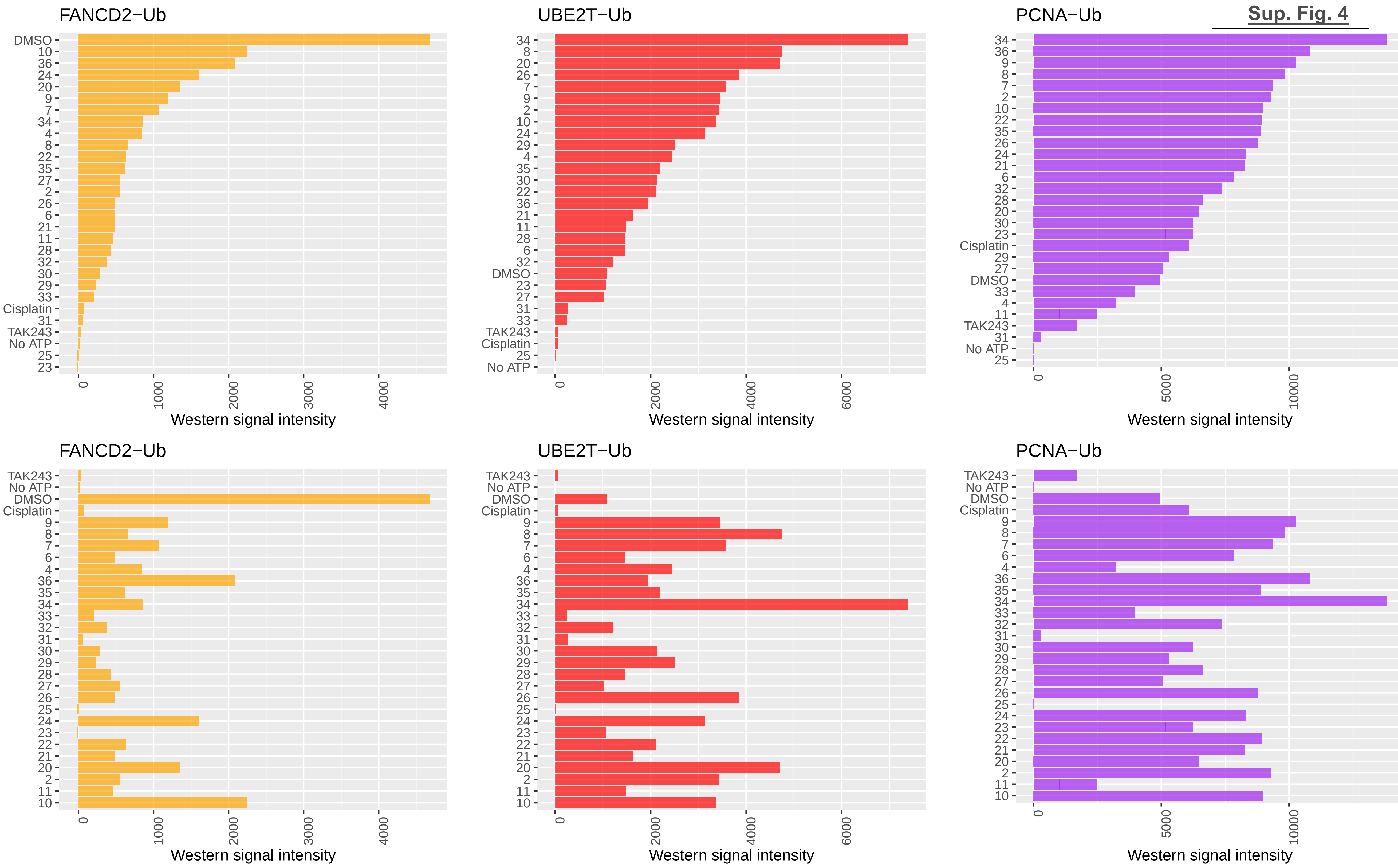
Sup. Fig. 4
FANCD2−Ub
UBE2T−Ub
PCNA−Ub
DMSO
Cisplatin
TAK243
No ATP
Western signal intensity

Sup. Table 1

Compound ID	IC50 (µM)	R^2
CI01	1.874	0.9068
CI02	8.288	0.7752
CI03	>100	NA
CI04	4.68	0.9788
CI05	24.65	0.9607
CI06	14.2	0.9376
CI07	3.616	0.9423
CI08	1.86	0.9721
CI09	5.93	0.7446
CI10	3.962	0.759
CI11	2.7	0.8789
CI12	0.4259	0.8198
CI13	>100	0.7572
CI14	>100	0.04846
CI15	13.39	0.8999
CI16	>100	0.3757
CI17	>100	0.4687
CI18	>100	0.7919
CI19	5.4	0.9166
CI20	18.01	0.8596
CI21	12.91	0.9906
CI22	7.363	0.8111
CI23	4.899	0.9105
CI24	8.067	0.8754
CI25	0.6111	0.9266
CI26	11.67	0.9755
CI27	5.738	0.977
CI28	13.58	0.4905
CI29	25.51	0.9819
CI30	7.685	0.9793
CI31	1.025	0.9758
CI32	5.248	0.8764
CI33	2.932	0.9559
CI34	4.262	0.9933
CI35	9.93	0.987
CI36	10.56	0.9952
